# The efficacy and safety of first-line anti-seizure medications as substitution therapy for children with drug-resistant epilepsy: a randomized controlled trial protocol

**DOI:** 10.3389/fneur.2023.1237183

**Published:** 2023-08-07

**Authors:** Roro Rukmi Windi Perdani, Wawaimuli Arozal, Irawan Mangunatmadja, Nastiti Kaswandani, Setyo Handryastuti, Bernie Endyarni Medise, Harri Hardi, Rajarajan Amirthalingam Thandavarayan, Hanifah Oswari

**Affiliations:** ^1^Department of Child Health, Faculty of Medicine Universitas Indonesia, Cipto Mangunkusumo Hospital, Jakarta, Indonesia; ^2^Department of Child Health, Faculty of Medicine, University of Lampung, Bandar Lampung, Lampung, Indonesia; ^3^Department of Pharmacology and Therapeutics, Faculty of Medicine, Universitas Indonesia, Jakarta, Indonesia; ^4^Department of Cardiovascular Sciences, Houston Methodist Research Institute, Houston, TX, United States

**Keywords:** antiepileptic drug, children, drug-resistant epilepsy, randomized controlled trial, substitution therapy

## Abstract

Although many anti-seizure medications (ASMs) are available, treatment failure, known as drug-resistant epilepsy (DRE), still occurs in around 30% of children with epilepsy. Second-line ASMs are usually used as substitution therapy in DRE to control seizures, although international consensus is not available yet. Previous studies focus on comparing the ASMs, whether as add-on or substitution therapy, mainly conducted in newly diagnosed epilepsy. However, the study that investigated first-line ASMs as substitution therapy compared to second-line ones, particularly among DRE children, is still lacking. A randomized controlled trial (RCT) enrolling 102 participants, aged 1–18, at three referral hospitals in Indonesia will be conducted, dividing them into intervention and control groups. The intervention group will be treated with first-line ASMs as the substitution therapy, while the other in the control group will get second-line ASMs. The primary outcome measure is the proportion difference of responders between groups who get first-line and second-line ASMs in 14 weeks of intervention.

**Clinical trial registration**: ClinicalTrials.gov, identifier NCT05697614.

## 1. Introduction

Epilepsy is the most prevalent chronic neurologic disorder in childhood, affecting 0.5 to 1 percent of children (1). According to the latest estimates, about 0.6% of children aged 0–17 years have active epilepsy (2, 3). In the first 3 years of life, around 35% of children with epilepsy develop DRE. The significant predictors are age ≤ 12 months at diagnosis, developmental delay at initial diagnosis of epilepsy, neuroimaging abnormality, and focal slowing on initial EEG (4). Epilepsy in children is caused by multifactorial, mostly hypoxic injury (38.71%) and unknown (32.26%) (5). Unknown etiology, which usually refer to specific genetical disorder, is usually hard to treat and often cause DRE (6).

The International League Against Epilepsy (ILAE) has defined DRE as the failure of therapy with two or more ASMs, single or combination therapy, to achieve seizure-free (7–9). Based on a meta-analysis, higher DRE was found in children (25.0%; 95% CI: 16.8, 34.4) than in adults (14.6%; 95% CI: 8.8, 21.6) (10). The term seizure-free refers to the absence of all seizure types, including aura (7). In children, an uncontrolled episode may impair brain development, cause behavioral disorders, and lower their quality of life (11–13).

The selection of appropriate ASMs for children is determined by seizure type, epilepsy type, epilepsy syndrome, epilepsy etiology, and comorbidities (14). There are algorithms for medication treatments for children with epilepsy, from administering the minimal therapeutic dose to providing add-on or substitution therapy. After a first-line ASM is administered and titrated, there is still a chance that the seizure is uncontrolled yet. If the seizure persists, additional or alternative first-line therapy is administered. Second-line ASMs are added after failing in the optimal dose of first-line ASMs with good compliance (15–17).

Despite the existence of a treatment algorithm, there has not been any international guideline for the management of DRE with combination therapy. Nonetheless, some regional recommendations are available. Other factors that may influence the seizure reduction are gender, age at onset, family history of seizure, previous history of febrile seizure or neonatal seizure, mental and motor retardation, seizure types, history of status epilepticus, presence of a specific epileptic syndrome or abnormal findings on EEG or brain imaging which may contribute to DRE (15, 18). Other supplementary nonpharmacological treatments are also considered effective, such as the ketogenic diet and Mozart’s music (19, 20).

The first-line ASMs commonly used for generalized epilepsy are valproic acid, phenobarbital, and phenytoin; carbamazepine is used for the focal type. These medications are categorized as older agents. On the other hand, newer agents such as topiramate, levetiracetam, and oxcarbazepine are classified as second-line ASMs (21, 22).

The American Academy of Neurology (AAN) subcommittee reported in 2004 that newer ASMs were no different in their capacity to control seizures but were significantly less neurotoxic and more tolerable (23–25). Numerous trials comparing the efficacy and safety of first- and second-line ASMs in children newly diagnosed with epilepsy have demonstrated that first-line agents are as effective as second-line agents. The seizure freedom rate in children with absence epilepsy treated with phenytoin did not differ from those with oxcarbazepine (26). Another study explained valproic acid, carbamazepine, and phenobarbital have the same efficacy as levetiracetam for newly diagnosed epilepsy in children (27). These results were in line with a study by James, which stated that both first-line agents (valproic acid and carbamazepine) and second-line agents (topiramate) are equally effective in treating newly diagnosed epilepsy in children (28). While for DRE, previous research investigated that children were not seizure-free even though they had been treated with levetiracetam as an add-on therapy (29).

Randomized studies in DRE typically involve the addition of new ASMs. However, in clinical practice, if a patient is already taking multiple medications, it is common to substitute one of the current medications despite the lack of evidence supporting this practice (30). The choice of the substitution drug depends on the mechanism of action, the pharmacokinetic profile, drug interaction, the risk of seizure exacerbation, and patient-related factors. The newer drug combination is intended to maximize efficacy and minimize toxicity. However, accessibility and cost-effectiveness should also be considered, as the patient would not acquire the optimal combination of drugs if they could not afford it or obtain the medication because it is unavailable in the hospital (31–34).

There are challenges in managing DRE in children in Indonesia, mainly where the study will be conducted. Like those in other countries, a study at Cipto Mangunkusumo National Central Public Hospital, Jakarta, showed that around 43 and 31% of children who were given second-line ASMs, topiramate or levetiracetam, respectively, did not achieve seizure remission (35). In addition, the number of medications covered by social insurance at Jakarta’s outpatient pediatric neurology clinic is limited, resulting in the additional needed drugs being fulfilled at their own expense. Moreover, the newer agents are sometimes unavailable even in rural referral hospitals.

Repeating the medication cycle can still be the option considering drug-resistant patterns where patients may initially be unresponsive to treatment but change into a treatment-responsive state. First-line ASMs can be regarded as substitution therapy since evidence has shown they have equal efficacy to second-line ones. However, there is still insufficient evidence and investigation for the therapeutic efficacy of first-line ASMs as a substitution for second-line ones. Therefore, this study aims to analyze the effectiveness and safety of first-line ASMs as substitution therapy in DRE children who have obtained second-line medication.

## 2. Methods and analysis

### 2.1. Objectives of the study

#### 2.1.1. Primary objective

The study’s primary outcome is to analyze the proportion of responders, defined as subjects who get 50% of seizure reduction. The proportion will be analyzed from eight to twelve weeks after the intervention.

#### 2.1.2. Secondary objectives

Some secondary objectives are to analyze the different improvements in quality of life, improvement of EEG feature, and time to achieve seizure reduction between the intervention group with first-line ASMs as drug substitution and the control group who gets second-line ASMs. The other objectives are to describe clinical characteristics, adverse drug reactions, and laboratory tests related to the adverse effects. Furthermore, this study will explain factors contributing to the reduction of seizure frequency and analyze the differences in responders based on the number of patient risk factors.

### 2.2. Design

This protocol is intended for a multicenter, open-label experimental, randomized controlled trial on the efficacy and safety of using first-line ASMs as substitution therapy in children with DRE. It will prospectively follow up on 102 children for 14 weeks in the baseline, intervention, and post-intervention phases. The study will be conducted at the pediatric outpatient clinic at three Jakarta referral hospitals: Cipto Mangunkusumo National Central Public Hospital, Harapan Kita Women and Children Hospital, and Fatmawati Central General Hospital. Children who were diagnosed with DRE and got levetiracetam or topiramate will be enrolled in the study.

### 2.3. Inclusion criteria

1. Children aged 1–18 years old.
2. Diagnosed with DRE by pediatric neurologists, based on the ILAE 2017 criteria (36).
3. Children with at least 3 months of combination therapy consisting of either levetiracetam or topiramate with an optimal therapeutic dose.

### 2.4. Exclusion criteria

1. Non-convulsive epilepsy.
2. Suffered from status epilepticus in the prior 3 months before the study
3. Past medical history of idiosyncrasies or severe adverse drug reactions caused by planned substitution therapy

## 3. Instrument of the study

### 3.1. Quality of life

Quality of life will be assessed by QOLCE-55 (Quality of Life in Childhood Epilepsy Questionnaire), a parent-reported and self-administered questionnaire that evaluates epileptic children aged 4–18 quality of life. It has 55 questions, including cognitive (22 items), emotional (17 items), social (7 items), and physical (9 items) functions. Items are rated on a five-point Likert scale, 0 = very often, 1 = fairly often, 2 = sometime, 3 = almost never, and 5 = never. The composite score is the unweighted average of the four subscales, ranging from 1 to 100. A higher score indicates better quality of life (37).

### 3.2. Diary card

Subjects’ adherence, seizure frequencies, and adverse drug effects will be evaluated by a self-reported diary card filled out by the parents each day during the intervention phase. In addition to measuring drug consumption, compliance will be measured by counting the tablets or powder packages at each study visit.

### 3.3. Electronic medical records

Secondary data, including demographic data, clinical characteristics, and imaging such as brain CT or MRI will be collected from Electronic Medical Records.

### 3.4. Laboratory test

The test is conducted twice in the hospital laboratory where the study is conducted, pre-and post-intervention. The blood test consists of a complete blood count, liver function test (ALT and AST), kidney function test (serum urea and creatinine), and blood electrolyte (sodium, potassium, and chloride). Verbal informed consent will be obtained from the participant’s parents or legal guardians.

### 3.5. Electroencephalography (EEG) examination

The EEG examination will be performed twice, at the baseline and post-intervention phase, using a high-density machine (Caldwell Easy III). The machine will operate for about 45 min, including 5 min each for opened-eye and closed-eye in every subject. EEG recordings use a standard parameter to analyze brain activity at various frequencies to gain good quality and artefact-free results.

## 4. Randomization

Patients will be randomized using a simple randomization method using computer-generated random numbers. Hence it is expected to get an equal number of participants among groups. One of our trial members will be assigned to perform randomization. After the table of sequence number of participants is available, they will be recruited by consecutive sampling technique.

## 5. Study procedure

**Figure 1:**
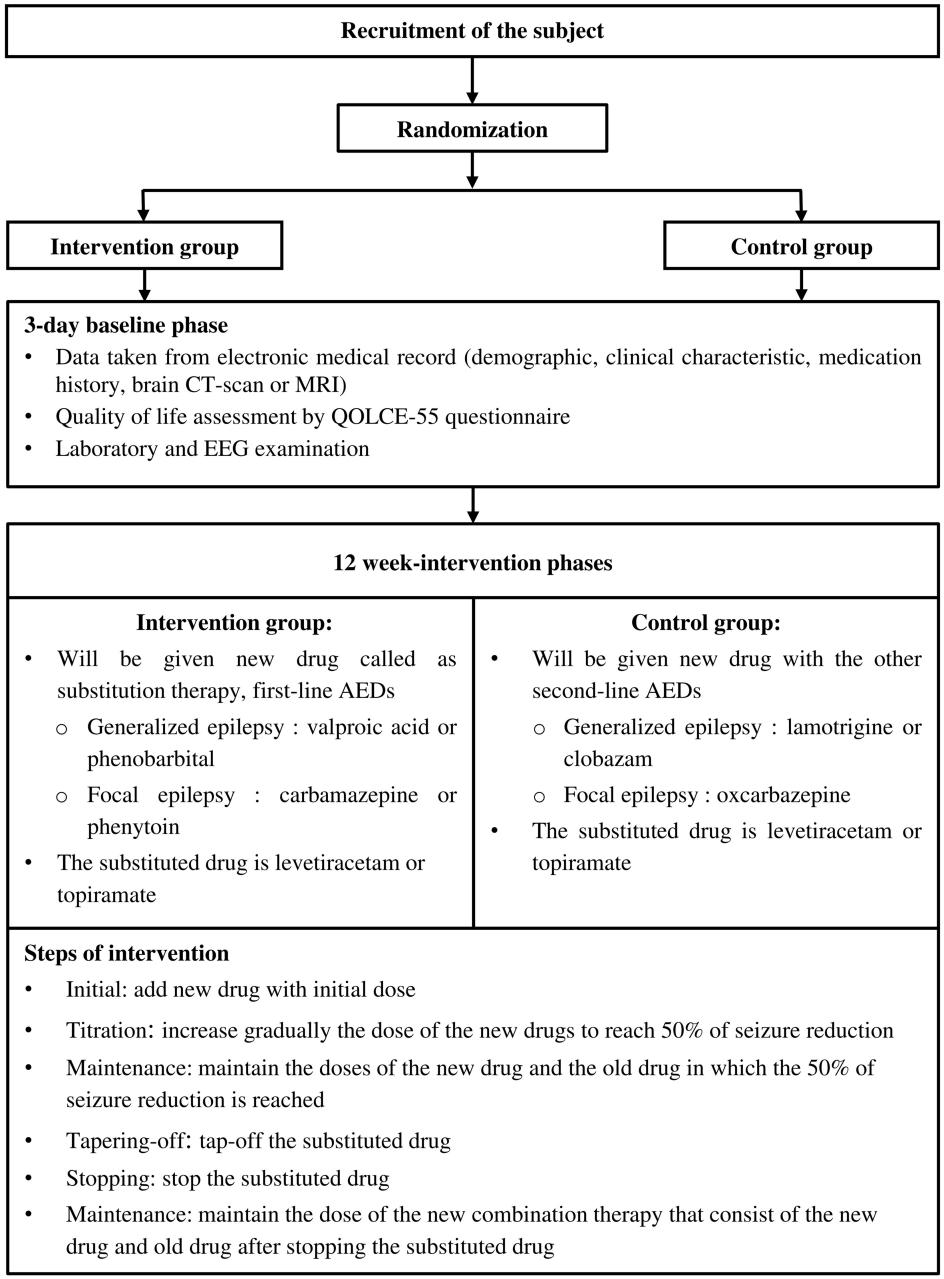
Flow diagram of subject recruitment, randomization, and steps of intervention.

The eligible patients who have given their consent will be enrolled. They will be divided into an intervention and control group before the 3-day baseline phase begins. In this phase, demographic and clinical characteristics, including seizure frequency, seizure type, age at onset, medication history, family history of seizure, and developmental stages, will be recorded from the electronic medical record along with a brain CT scan or MRI result. After that, their quality of life will be assessed by QOLCE-55 validated questionnaire through a self-guided report. Furthermore, laboratory investigation and EEG will be performed. The study procedure is described in Figure 1.

**Figure 2:**
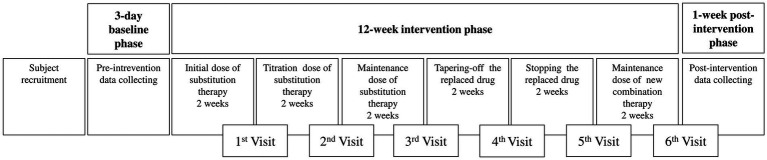
Detailed intervention phase and its monitoring.

The next phase is the intervention phase, which starts from the initial step and ends with the maintenance of new combination therapy. It will be divided into six steps, as described in Figure 2. Initially, the substitution drugs with each initial dose will be consumed. The phase continues with titration dose, where the drug dose will be gradually adjusted until it causes 50% of seizure reduction, and the next step is to maintain the dose for about 2 weeks.

The following phase is tapering off and stopping the substituted drug, determined by individual condition. Nonetheless, if the frequency of seizures increases by more than 1.5 times during these phases, the intervention will be terminated immediately. Nonetheless, if seizures frequency increases by more than 1.5 times during these phases, the intervention will be terminated immediately. On the contrary, if the condition is improved (seizure frequency does not increase or there is reduced seizure frequency compared to the baseline state), the subject will maintain the new drug combination phase consisting of the substitution and the old drugs.

Subjects are categorized as non-responders if seizure frequency (1) increases, (2) remains the same, or (3) decreases but not up to 50% of the baseline when the intervention is ended. Non-responders will be given the former combination therapy with adjusted doses to decrease the seizure frequency until reaching the pre-intervention condition.

The dropout criteria are resignation, non-compliance with study procedure, death in terms of other causes unrelated to the study, and the patient did not enter the study until at least the tapering-off phase. The lost to follow-up term is intended for those not attending the study without any possible reasons.

## 6. Sample size estimate

The proportion of seizure reduction on standard drugs was found at 70% (38). We expect a 25% seizure proportion difference as clinically significant between the new treatment drug and the control. The estimated sample size in this study was calculated using the different proportion formula for the two unpaired groups, resulting in a sample size of 51 in each group (102 in total) after considering 10% dropout with 80% power and 5% significant level.

## 7. Statistical analyzes

Intention-to-treat analysis will be used to interpret the data, but per-protocol analysis will also be considered to be analyzed if any significant differences exist. Data will be analyzed by IBM SPSS Statistics version 22.

All the categorical demographic data will be shown in frequency and percentage. In contrast, numerical demographic data will be shown in mean ± standard deviation or median (min-max) based on Kolmogorov–Smirnov normality test. ARR (absolute risk reduction), which stands for the difference in increased seizure proportion between the control and intervention group, and NNT (number needed to treat) will also be assessed to recognize the effectiveness of the drug regimen on the participants.

Chi-square will be used to analyze the differences in the proportion of responders and EEG appearance if the normality assumption was satisfied; otherwise, Fisher’s multiple tests will be used. The differences between the mean quality of life and time to achieve seizure reduction will be analyzed with the Unpaired T-test or Mann–Whitney, whichever is appropriate.

Multivariate logistic regression analysis will be performed to analyze any factor that contributes to seizure reduction (current age, age at onset of epilepsy, seizure type, family history of seizures, developmental delays, history of ASM, initial seizure frequency, initial EEG, brain structural abnormalities) in both intervention and control groups. A dummy table for this statistical analysis is also provided in Table 1.

**Table 1:** Dummy table for multivariate analysis.

Variable	Univariate		Multivariate		
	OR (95% CI)	*p*-value	Coefficient (β)	aOR (95% CI)	*p*-value
Age					
0–5					
5–12					
12–18					
Age of epilepsy onset					
0–5					
5–18					
Gender					
Male					
Female					
Family history					
Yes					
No					
Developmental delay					
Yes					
No					
Seizure type					
Focal					
Generalized					
Initial seizure frequency					
<15 min					
≥15 min					
EEG abnormalities					
Yes					
No					

Variables which hypothesized contributed to seizure reduction will be evaluated using bivariate analysis. The bivariate predictors with *p*-value < 0.20 will be exported to the multivariate logistic regression model.

## 8. Discussion

Young children may become more hypersensitive to AEDs due to age-related changes in drug metabolism, i.e., the higher rate of CYP-mediated responses. For instance, children are ten times more likely than adults to experience mild or severe rashes when exposed to lamotrigine (39). Furthermore, children under five are three to five times more likely than adults to develop SCARs (severe cutaneous adverse reactions) and other rash associated with AEDs (40). Lamotrigine (45.4%) and carbamazepine (45.4%) were the AEDs most frequently implicated in hypersensitivity responses in children (41). Considering these studies, we will exclude potentially developing AED hypersensitivity patients. We also will educate the subjects and their parents to inform us if they develop any signs and symptoms of hypersensitivity. We will also examine the possibility of any drug hypersensitivity at every visit. The subject will be dropped out if it happens. To our knowledge, no standard guidelines are available for managing drug-resistant epilepsy in children. There is insufficient evidence to show the superiority of second-line ASMs compared to first-line regarding drug-resistant epilepsy in children, even though the safety profiles are proven to be better. In addition, the availability of the second line ASMs, particularly in remote area hospitals, is often not fulfilled.

Alternative therapy, such as a ketogenic diet, is also promising but expensive and requires the management of a trained nutritionist (19). Mozart’s music is another intervention that is seen to be effective (42). However, a meta-analysis showed that studies related to Mozart’s music are still few, and the most effective protocols for therapeutic potential need further definition (20). Since numerous factors influence the DRE mechanism, repeating the medication cycle is still possible when considering drug-resistant patterns in which initially treatment-unresponsive states may be transformed into treatment-responsive.

We hope that by sharing this protocol, other researchers can perform similar studies and get more comprehensive results. Hopefully, this study result will become a reference for clinicians’ clinical practice, especially in developing countries.

## Ethics statement

The studies involving humans were approved by Faculty of Medicine Universitas Indonesia. The studies were conducted in accordance with the local legislation and institutional requirements. Written informed consent for participation in this study was provided by the participants' legal guardians/next of kin.

## Author contributions

RP, WA, IM, NK, BM, and RT concepted and designed the study. RP, WA, and HH wrote the first draft of the manuscript. HO supervised the trial and analyzed the data. All authors contributed to the article and approved the submitted version.

## Funding

This research was funded by the grant of PUTI Q2 2022/2023, Universitas Indonesia, with the grant number: NKB-1424/UN2.RST/HKP.05.00/2022.

## Conflict of interest

The authors declare that the research was conducted in the absence of any commercial or financial relationships that could be construed as a potential conflict of interest.

## Publisher’s note

All claims expressed in this article are solely those of the authors and do not necessarily represent those of their affiliated organizations, or those of the publisher, the editors and the reviewers. Any product that may be evaluated in this article, or claim that may be made by its manufacturer, is not guaranteed or endorsed by the publisher.
